# How Frequently are Caregivers Included in Patient Education for Oncology Patients?

**DOI:** 10.1093/geroni/igab046.2932

**Published:** 2021-12-17

**Authors:** Daniel Hekman, Anne Mueller, Beth Fields

**Affiliations:** University of Wisconsin-Madison, Madison, Wisconsin, United States

## Abstract

A growing body of literature shows that family and unpaid caregivers of older adults with cancer are assuming more care responsibilities, especially after discharge from an inpatient admission, and frequently report feeling unprepared to do so. Interprofessional collaborative practice can rectify this gap to help ensure caregivers are included in the care team and patient education in the hospital. This retrospective data analysis of electronic health record data examines the prevalence of caregiver involvement in education activities conducted by health care practitioners for older adult cancer inpatients at an academic medical hospital in the midwestern United States. Our dataset includes a total of 676 admissions of older adult cancer inpatients (565 unique patients) between 9/1/2018 and 10/1/2019. Descriptive statistical analyses were conducted to determine the prevalence of caregiver involvement in patient educational activities. The average patient was 75 years old (range: 66-89), white(95%) and male (57%). Approximately 5,720 educational topics were discussed with patients, and 88% of admissions included some patient education. Caregivers were included in 29.6% of educational topics discussed and at least one education session for 42.9% of all admissions. Caregivers are important collaborators in supporting the health and well-being of older adults with cancer, but they are often not included in patient educational activities prior to discharge. Practioners may need to evaluate barriers to including caregivers in patient education activities. A better understanding of this gap in education can help inform future interprofessional collaborative practice initiatives.

